# Antibiotic Resistance Patterns of Diverse *Escherichia coli* Phylogenetic Groups Isolated from the Al-Hillah River in Babylon Province, Iraq

**DOI:** 10.1155/2019/5927059

**Published:** 2019-09-02

**Authors:** Mourouge Saadi Alwash, Hawraa Mohammed Al-Rafyai

**Affiliations:** Department of Biology, College of Science, University of Babylon, Hillah, Iraq

## Abstract

Surface water contamination remains a major worldwide public health concern and may contribute to the dissemination of antibiotic-resistant bacteria. The Al-Hillah River in the city of Babylon Province, Iraq, diverts flows from the Euphrates River. Because of its importance in irrigation and population density, it faces several forced and unforced changes due to anthropogenic activities. To evaluate water quality, water samples were collected from three sites with different anthropogenic pressures along the Al-Hillah River. These samples were subjected to bacteriological analyses, i.e., total coliforms, *Escherichia coli*, and faecal enterococci. The phylogenetic groups of the *E. coli* isolates (*n* = 61) were typed by rapid PCR-based analyses. Representatives of each isolate were tested phenotypically for resistance to six classes of antibiotics and characterized according to their phylogenetic groups. The results demonstrated the highest resistance levels were to *β*-lactam antibiotics, followed by fosfomycin and aminoglycosides. *Escherichia coli* isolates belonging to phylogenetic groups A and B2 were the most common and were characterized by a higher prevalence of antibiotic resistance. This study is important for understanding the current conditions of the Al-Hillah River, as the data reveal a high prevalence of multiresistance among *E. coli* isolates circulating at the three sampling sites.

## 1. Introduction

Water is an essential component of life. Management of water is the key to ensuring its efficient and equitable use and to encouraging conservation of water resources [1, 2]. The release of by-products of anthropogenic activities (due to expanding human populations, intense iagricultural activities, and discharges of untreated sewage wastewater) into the river water is a major source of inputs, leading to increased deterioration of water quality [3]. Thus, consumption of this contaminated water raises the danger of exposure to enteric bacterial, viral, and protozoan pathogens that can cause severe diseases in people who use the water for recreational activities, fishing, drinking, bathing, and crop, especially from those products eaten raw [4, 5]. An additional danger is that untreated wastewater effluent is considered a significant carrier of antibiotic resistance (AR) determinants [6]. The dissemination of AR among pathogenic bacteria is a serious threat in the natural environment. AR may occur either by mutation or acquisition of antibiotic resistance genes (ARGs) through horizontal gene transfer (HGT). In aquatic environments, HGT is one of the major mechanisms used to spread ARGs from environmental and commensal species to pathogenic ones [7, 8]. Thus, rivers can be efficient vehicles for the dissemination of AR [9, 10].

*Escherichia coli* is a part of the intestinal flora in humans and warm-blooded animals and is frequently used as a faecal indicator to monitor the microbial quality of water sources [11]. *Escherichia coli* isolates have been categorized into (i) commensal, (ii) intestinal pathogenic, or (iii) extraintestinal pathogenic [12]. Besides, *E. coli* isolates mainly fall into four phylogenetic groups, A, B1, B2, and D, based on combinations of three genetic markers: (i) *chu*A, a gene, that is responsible for haeme transport in enterohaemorrhagic O157 : H7 *E. coli*; (ii) *yja*A, a gene of unknown function that was identified in the recent complete genome sequence of *E. coli* K-12; and (iii) TspE4.C2, an anonymously designated DNA fragment of a noncoding region in *E. coli* isolates [13]. Commensal *E. coli* isolates are commonly associated with phylogenetic groups A and B1, while the extraintestinal (noncommensal) pathogenic *E. coli* isolates belong mostly to the B2 phylogenetic group and (to a minor extent) to group D. Commensal *E. coli* isolates do not normally carry any known virulence factors [14]. In contrast, pathogenic *E. coli* isolates carry virulence-associated factors that are commonly associated with extraintestinal infections [13, 14].

It is crucial that we improve our understanding of the Al-Hillah River habitat due to its importance for community livelihoods. A literature search shows gaps in the previous studies. Earlier studies were not comprehensive in their analysis of the microbial properties [15]. Furthermore, the data are about five or more years old; therefore, knowing the status of the pollution in the Al-Hillah River is important, especially due to increased anthropogenic activities in that region. In addition, studies highlighting *E. coli* phylogenetic groups may play crucial roles in the monitoring the water's pollution sources. To date, there is no published study on the phylogenetic variability of the *E. coli* isolates recovered from the Al-Hillah River. Therefore, the aim of this study was to disclose AR and use molecular characterization to reveal the diversity of the phylogenetic groups among the *E. coli* isolates recovered from the Al-Hillah River in Babylon Province, Iraq.

## 2. Materials and Methods

### 2.1. Study Area Description

**Figure 1:**
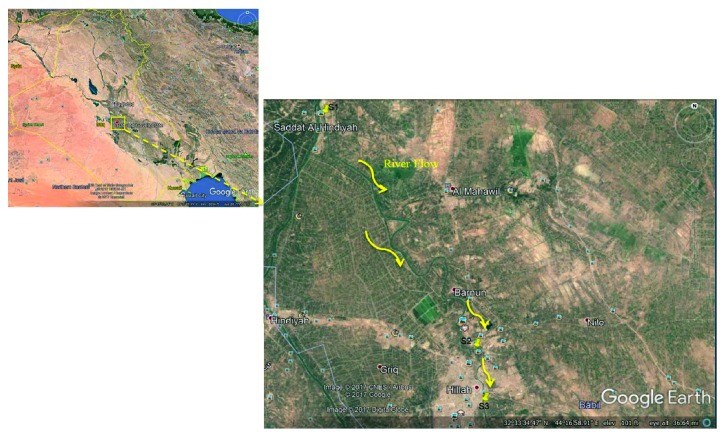
Locations of three sampling sites (S1–S3) along the Al-Hillah River, Babylon province, Iraq.

The present study was conducted along the Al-Hillah River in Al-Hillah city, which is located in the province of Babylon, Iraq. The river is situated at a latitude of 32°33′N and a longitude of 44°45′E and at an altitude of 4–4.9 m above the mean sea level (Arabian Sea) (Figure 1). The length of this river is approximately 97 km. Many pollution sources are distributed randomly near this river, such as agricultural sites, sewage draining facilities, and discharges pipes from drinking water purification stations. People living in rural areas depend directly on this river to supply water for irrigation and domestic activities due to the irregular provision of water supply throughout the country. The sampling areas were selected to include sites influenced by diverse sources of human activities. One sampling site (S1) is located in an intensive agriculture area; second site (S2) is located near Marjan for Internal Medicine and Cardiology Hospital; and the third site (S3) corresponds to urbanized areas. Seventy-five water samples were collected from each site along the Al-Hillah River in mid-December 2017.

### 2.2. Isolation and Enumeration of *E. coli* and Other Pathogens

Water samples (0.5 m depths) were collected from three sites in sterile glass bottles (1000 mL). All samples were stored in a cooler box with ice packs, immediately transported into the laboratory, and kept at 4°C until they were analysed within 24 hours (h) of sampling. Membrane filtration technique was used to isolate faecal indicators [16]. Briefly, water samples (100 mL) were filtered through 47 mm membrane filters (Cellulose Nitrate filter, Sartorius Stedium Biotech GmbH, Göttingen, Germany) with a nominal pore size of 0.45 *μ*m using a vacuum filtration system. Following filtration, total coliform, *E. coli*, and enterococci were detected and enumerated using Chromogenic coliform, Hi-Crome *E. coli*, and Slanetz and Bartley media (HiMedia Laboratories Prt. Ltd, Mumbai, India), respectively. Counts were recorded as CFU/mL. Hi-Crome *E. coli* plates were incubated at 37°C for 24 h. After incubation, blue colonies were counted as presumptive *E. coli*. *E. coli* isolates then picked and purified on Brain Heart Infusion medium. After purification, presumptive *E. coli* isolates were kept in slant and in glycerol forms (Brain Heart Infusion Broth with 50% glycerol) at 4°C and −20°C, respectively, for further analysis. Isolates were confirmed as presumptive *E. coli* using Enterosystem 18R (Liofilchem® S.r.l., Italy), according to the manufacturer's instructions. Slanetz and Bartley plates were incubated at 37°C for 24–48 h. Isolates that were catalase negative and able to grow in 6.5% NaCl and to hydrolyze esculin in the presence of 40% bile salts were considered as presumptive *Enterococci*.

### 2.3. Detection of Antibiotic Resistance among *E. coli* Isolates

An antibiotic susceptibility assay was utilized to determine the prevalence of antibiotic-resistant *E. coli* from the sampled isolates. A total of 15 antibiotics (Biomaxima, Poland), belonging to 6 classes, were assayed, including aminoglycosides: amikacin (AK, 10 *μ*g), gentamicin (CN, 10 *μ*g), and streptomycin (S, 10 *μ*g); tetracyclines: tetracycline (TE, 30 *μ*g) and doxycycline (DO, 30 *μ*g); *β*-lactams: ampicillin (AMP, 10 *μ*g), imipenem (IMP, 10 *μ*g), cephalothin (KF, 30 *μ*g), cefoxitin (FOX, 30 *μ*g), cefotaxime (CTX, 30 *μ*g), and cefepime (FEP, 30 *μ*g); fluoroquinolones: norfloxacin (NOR, 10 *μ*g) and ciprofloxacin (CIP, 10 *μ*g); fosfomycin (FF, 200 *μ*g); and phenicol: chloramphenicol (C, 30 *μ*g). The disk diffusion method was used to determine the AR patterns among the *E. coli* isolates. 24 h old pure cultures were subcultured in nutrient broth (NB; Himedia, India) and then incubated for 3 to 6 h at 37°C to achieve log phase growth. Next, the turbidity was adjusted in 0.85% sterile normal saline solution to 0.5 McFarland's standard [10^8^ (colony forming unit) CFU/mL] and aliquots were then spread on Mueller–Hinton agar (MHA; Himedia, India) with a sterile cotton swab. Antibiotic disks were placed onto the MHA inoculated with the bacteria and gently pressed down to ensure complete contact with the agar, and the plates were then incubated for 24 h at 37°C. The bacterial isolates were designated as resistant, intermediate, and susceptible as recommended by the Clinical Laboratory Standards Institute [17]. Resistant and intermediate isolates of *E. coli* were classified as nonsusceptible, while sensitive isolates were classified as susceptible. Multiple drug resistance (MDR; nonsusceptible to ≥1 agent in ≥3 antimicrobial categories) and the multiple drug resistance indices (MDRIs) of the isolates were estimated as previously described by Krumperman [18]. The MDR index (MDRI) = a/(b × c), where a is the aggregate antibiotic resistance score of isolates; b is the number of antibiotics, and c is the number of isolates.

### 2.4. Extraction of Genomic DNA

Genomic DNA was extracted from *E. coli* after 24 h of incubation. The DNA extraction was carried out using the Favor Prep™ Genomic DNA Mini Kit (Favorgen, Taiwan). The DNA quality and quantity were assessed using a NanoDrop spectrophotometer (Implen, Germany). Genomic DNA was extracted in duplicate from each independent sample. The genomic DNA samples were stored at −20°C until further analysis.

### 2.5. Determination of Phylogenetic Groups

All isolates were typed to one of the four major *E. coli* phylogenetic groups (A, B1, B2, and D) according to Clermont et al. [13] via a polymerase chain reaction-based assay (PCR) that evaluated the genetic markers *chuA* and *yjaA* and the TspE4.C2 DNA fragment. The reaction mixture (20 *μ*L) contained 2 × master mix (5 *μ*L), 10 *μ*M forward and reverse primers (4 *μ*L, 2 *μ*L each), Genomic DNA (4 *μ*L), and RNase-free water (7 *μ*L). The PCR programme was as follows: 2 minutes (min) at 50°C, 4 min at 95°C, followed by 30 cycles for 30 second (sec) at 94°C, 59 cycles for 30 sec at 53°C, and 30 cycles for 30 sec at 72°C. The amplification products were separated in 2% agarose gel containing ethidium bromide. After electrophoresis, the gel was visualized and photographed under UV light. The isolates were assigned to the four main phylogenetic groups A (*chuA*−, TspE4.C2−), B1 (*chuA*−, TspE4.C2+), B2 (*chuA*+, *yjaA*+), and D (*chuA*+, *yjaA*−). To increase the resolution of the isolate discrimination, subgroups were determined as follows: subgroup A_0_ (group A), *chuA*−, *yjaA*−, TspE4.C2−; subgroup A_1_ (group A), *chuA*−, *yj*a*A*+ TspE4.C2−; group B1, *chuA*−, *yjaA*−, TspE4.C2+; subgroup B2_2_ (group B2), *chuA*+, *yjaA*+, TspE4.C2−; subgroup B2_3_ (group B2), *chuA*+, *yjaA*+, TspE4.C2+; subgroup D_1_ (group D), *chuA*+, *yjaA*−, TspE4.C2−; and subgroup D_2_ (group D), *chuA*+, *yjaA*−, TspE4.C2+ [19].

### 2.6. Statistical Analysis

Pearson correlation coefficients were used to analyse the relationships among the bacteriological water quality parameters using the Statistical Package for the Social Sciences (IBM SPSS Statistics for Windows, Version 20.0 Armonk, NY). Statistical significance was defined as *p* ≤ 0.05. Principal component analysis (PCA) was utilized to describe the distribution of the *E. coli* isolates with respect to their phylogenetic groups and AR patterns at the three sampling sites. PCA was performed with the R Statistical Package for Windows version 3.4.2. To perform the PCA procedure, a data set of possibly correlated variables was transformed into a set of values for linearly uncorrelated variables called principal components. This transformation is defined in such way that the first principal component (PC1) captures the maximum variance and direction in the data set, whereas the second principal component (PC2) captures the remaining variance in the data set and is uncorrelated to the PC1 components (R Development Core Team, 2008).

## 3. Results

### 3.1. Distribution of *E. coli* and Other Pathogens

The bacteriological parameters in the water samples collected from the three sites are presented in Table 1 as geometric mean values ± standard deviation. Currently, there is no enforceable water quality legislation in Iraq to which designated surface waters must comply; thus, based on the EPA advisory limits [20], most of the bacteriological parameters, including the total counts of coliform bacteria, *E. coli*, and *Enterococci*, were above the standard limits at the three sites. The highest and lowest total coliform counts were observed in the samples collected from S1 (4.7 × 10^3^–3.6 × 10^3^ CFU/100 mL), followed closely by S2 (4.6 × 10^3^–3.4 × 10^3^ CFU/100 mL), and S3 (4.6 × 10^3^–3 × 10^3^ CFU/100 mL). For the *E. coli* populations at S1, S2, and S3, the counts ranged from 3.5 × 10^3^ to 3 × 10^3^ CFU/100 mL, 3.4 × 10^3^ to 2.9 × 10^3^ CFU/100 mL, and from 3.3 × 10^3^ to 2.8 × 10^3^ CFU/100 mL, respectively. The highest level of *Enterococci* (0 to 1.9 × 10^3^ CFU/100 mL) was observed at the S1 site, which is located near agricultural lands, followed by S2 (0 to 1.6 × 10^3^ CFU/100 mL), and S3 (0 to 1.3 × 10^3^ CFU/100 ml). Table 1 also shows how the bacterial parameters correlate with each other. From the obtained data, increasing densities of total coliform counts in the water samples corresponded with increasing *E. coli* densities (*r* = 1.000, *p*=0.01).

### 3.2. Diversity of the *E. coli* Isolates

**Figure 2:**
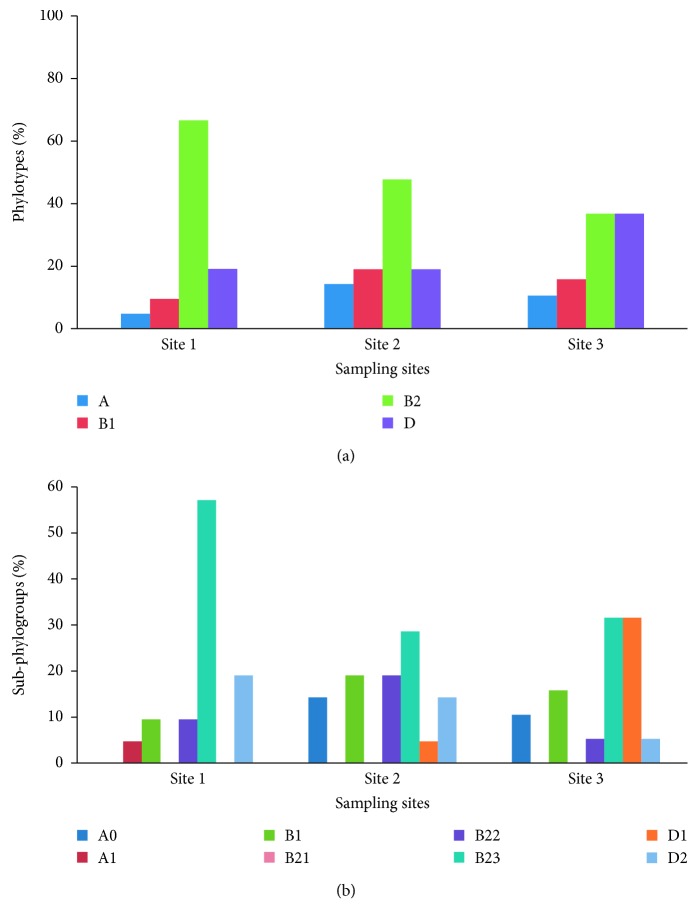
Distribution of the *E. coli* phylogenetic groups among the three sampling sites located along the Al-Hillah River. Distribution of *E. coli* (a) according to the phylogenetic groups (A, B1, B2, D) and (b) according to phylogenetic groups subtyping (A_0_/A_1_, B2_2_/B2_3_, and D_1_/D_2_).

A total of 61 *E. coli* isolates obtained from three sites were selected for further analyses. PCR was performed to analyse the *E. coli* diversity using the *chuA* and *yjaA* genes and the TspE4.C2 DNA fragment marker. Representative data of each unique PCR profile (*n* = 61) were then assigned to one of the main phylogenetic groups: A, B1, B2, and D. Group B2 was the most prevalent (31 isolates, 50.8%), followed by groups D (15 isolates, 24.6%) and B1 (9 isolates, 14.8%). The least prevalent group was A (6 isolates, 9.8%). For each site, group B2 was the most prevalent, followed by group D, while the least prevalent group was A (Figure 2(a)). The subgroups of each phylogenetic group (A_0_/A_1_, B2_2_/B2_3_, and D_1_/D_2_) were not distributed homogenously in three sites. Subgroup B2_3_ comprised almost more than half of the phylogenetic group B2 at site S1, while B2_2_ was the least prevalent subgroup observed at the three sites. Subgroup D1 was only detected at the S2 and S3 sites, which are both located in areas of anthropogenic pressure (Figure 2(b)).

### 3.3. Pattern of Antibiotic Resistance

**Figure 3:**
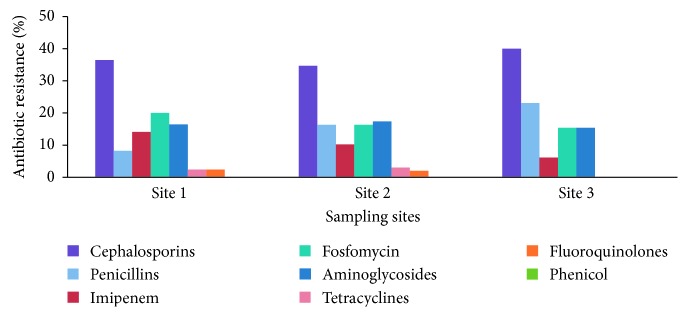
Distribution of antibiotic resistance prevalence among the three sampling sites.

Antibiotic resistance patterns were demonstrated for 61 *E. coli* isolates. All of the isolates were resistant to at least three agents in the six classes of antibiotics assayed. *β*-lactam resistance was most common (63.1%), followed by fosfomycin resistance (17.2%) and aminoglycoside resistance (16.4%). The isolates were more susceptible to tetracyclines (1.8%) and fluoroquinolones (1.5%). Differences in the proportions of resistance levels to the different antibiotic classes existed at all of the sampling sites are presented in Figure 3. Low levels of tetracycline and fluoroquinolone resistance were recorded at the S1 and S2 sites, and no resistance was detected at the S3 site. Furthermore, imipenem resistance was highly prevalent in the S1 and S2 sites compared with the S3 site (Figure 3). The MDR indices were 0.31, 0.27, and 0.23 for S2, S1 and S3, respectively (data not shown).

**Figure 4:**
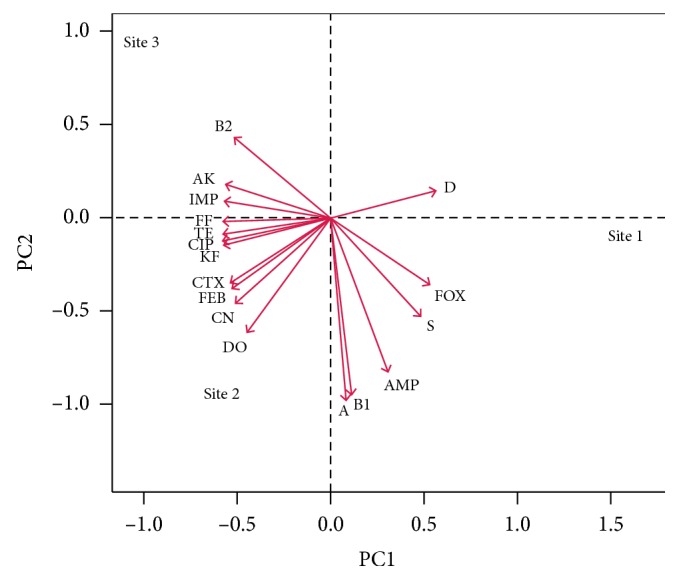
Principal component analysis biplot of the AR patterns of the *E. coli* phylogenetic groups from the three sampling sites along the Al-Hillah River. The red arrows indicate *E. coli* isolates resistant to 15 antibiotics with respect to their phylogenetic groups. Class I, CN, aminoglycosidase: gentamicin; AK: amikacin; S: streptomycin, Class II, TE, tetracyclines: tetracycline; DO: doxycycline, Class III, AMP, *β*-lactams: ampicillin; IMP: imipenem; KF: cephalothin; FOX: cefoxitin; CTX: cefotaxime; FEP: cefepime, Class IV, NOR, fluoroquinolone: norfloxacin; CIP: ciprofloxacin, Class V, FF: fosfomycin, Class VI, C, phenicol: chloramphenicol.

Principal component analysis (PCA) revealed significant differences among the sites with respect to their AR patterns. In comparisons among the sites, high AR was detected in the *E. coli* isolates recovered from the S2 site near Marjan for Internal Medicine and Cardiology Hospital (positive direction of PC2, Figure 4). The analysis of the phylogenetic groups in terms of their AR patterns indicated that phylogenetic groups A and B1 exhibited some level of resistance to antibiotics at the S1 site. However, the *E. coli* isolates in the A and B1 phylogenetic groups (positive direction of PC1) generally showed gradients of AR compared with those in phylogenetic group D (positive direction of PC1). The phylogenetic group B2 isolates demonstrated high levels of amikacin and imipenem resistance at the S3 site (positive direction of PC2).

**Figure 5:**
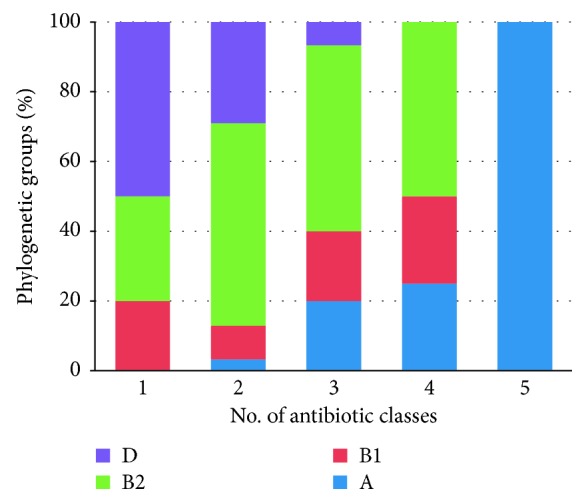
Distribution of the phylogenetic groups based on the number of antibiotic resistance patterns in *E. coli*.

Figure 5 summarizes the distribution of AR over the phylogenetic groups. Isolates belonging to phylogenetic groups A, B2, and B1 showed high level of resistance to the assayed antibiotics compared with those in group D. The majority of the *E. coli* isolates were MDR (resistance to ≥1 agent in ≥3 antibiotic categories).

## 4. Discussion

The release of manure effluents and sewage wastewater containing different bacterial pathogens into aquatic environments are a leading cause of the deterioration of aquatic resources [21]. Faecal bacteria, especially *E. coli*, are used as an indicator of possible pathogen presence in surface water due to its ability to persist in aquatic environments for a considerable period of time [22]. Most of the values of the microbial parameters obtained from the three Al-Hillah River sites were above the advisory limits [20], suggesting a high level of contamination by *E. coli* isolates at the studied sites; thus, the water from these sites should not be used for drinking, fishing, recreational activities, irrigation, or other purposes due to high risks to human health. This observation emphasizes the appropriateness of *E. coli* concentration as an indicator for monitoring the water quality in the Al-Hillah River.

The PCR assay developed by Clermont and colleagues [13] was used to detect the different phylogenetic groups. The highest affiliation rates of the *E. coli* isolates were in phylogenetic groups B2 (50%) and D (24.6%), which tend to contain more pathogenic isolates compared with groups B1 (14.8%) and A (9.8%) [23, 24]. According to the literature, commensal *E. coli* isolates are generally affiliated with groups A and B1 [14, 25], while noncommensal (extraintestinal) *E. coli* isolates are predominantly found in groups B2 and D [24, 26]. It should be noted, however, that isolates belonging to phylogenetic groups A and B1 have been more frequently isolated from aquatic environments than isolates belonging to phylogenetic groups B2 and D [27]. Ghaderpour et al. [6] reported that phylogenetic groups A and B1 were predominant in water samples collected from the Matang mangrove estuaries in Malaysia. Pereira et al. [10] found that isolates belonging to phylogenetic groups A and B1 were more prevalent than isolates belonging to groups B2 and D in the Tagus estuary in Portugal. Our findings, however, are in contrast with previous studies that showed that isolates belonging to the B2 and D phylogenetic groups were predominant in the Al-Hillah River. Thus, the Al-Hillah River might present a potential risk for exposure to pathogenic *E. coli* due to high prevalence of the B2 and D groups. Furthermore, a high proportion of the detected isolates belonged to phylogenetic group B2, especially to the B2_3_ subgroup. Carlos et al. [28] reported that the B2_3_ subgroup was present only in human faeces and that it could be a good indicator for human faecal pollution in aquatic environments. Overall, the isolates detected at the three sites were influenced by human faeces contamination; thus, identifying this kind of contamination is necessary for monitoring the bacteriological quality of water resources.

The emergence and dissemination of AR is showing an increasing trend among enteric bacteria [8]. The transfer of resistance among microorganisms is a serious threat that contributes to the development and emergence of AR, thereby reducing the therapeutic potential of antibiotics against pathogens [29]. Several studies have addressed the relationship between *E. coli* isolates and the prevalence of AR patterns in aquatic environments [10, 30]. The Al-Hillah River showed a high prevalence of antibiotic-resistant *E. coli* isolates with MDR rates of 80.3%, which is much higher than those observed in the Matang mangrove estuaries in Malaysia (34%) [6], the Tagus estuary in Portugal (19%) [10], and the Seine River in France (39%) [31]. However, the prevalence of MDR *E. coli* isolates in the Al-Hillah River was lower compared to that recorded in the Dongjiang River catchment in China (88%) [9]. As a result, high levels of AR in *E. coli* isolates reported in earlier studies from different regions and those reported in the present study might highlight the potential risk of AR dissemination in aquatic environments.

The highest prevalence of AR in *E. coli* was reported against the following antibiotics (in decreasing order): the *β*-lactams, including cephalosporins, penicillins, and imipenem, followed by fosfomycin and the aminoglycosides (Figure 3). The major AR patterns of the *E. coli* isolates observed in the present study were common in other aquatic environments [6, 10, 31–33]. These resistance patterns are realistic since these antibiotics, as well as others, can be easily purchased over the counter in Iraq with an accompanying general lack of education and awareness. Furthermore, these antibiotics are widely used as growth promoters in animal farming and for other agricultural purposes. Thus, sewage discharges and manure effluents may contain antibiotics and AR determinants that contaminate natural aquatic environments [34]. Last resort *β*-lactam antibiotics, such as imipenem, are reserved for patients with difficult to manage infections for which other *β*-lactams and aminoglycosides are not effective. The prevalence of imipenem-resistant *E. coli* isolates at the three sites indicates the role of the natural aquatic environment as a reservoir and disseminator of ARGs. Furthermore, this finding is likely due to the anthropogenic selective pressures imposed by the release of antibiotics and/or AR determinants found in clinical environments. Therefore, caution is urgently needed to prevent inappropriate and indiscriminate use of antibiotics as medications and for other prophylactic purposes, especially in developing countries such as Iraq, where the drugs can be obtained without prescription due to lack of drug regulation [35].

Varying distributions of AR over the *E. coli* phylogenetic groups have been reported in numerous studies. Bukh et al. [36] and Mosquito et al. [30] found that phylogenetic group D had the highest prevalence of MDR. Garcia-Aljaro et al. [37] and Pereira et al. [10] reported a lower prevalence of MDR in isolates in phylogenetic groups B2 and B1, whereas Ghaderpour et al. [6] found that the highest MDR prevalence was in isolates belonging to phylogenetic groups A and B1. In the present study, phylogenetic group A was distinguished by a higher MDR prevalence, followed by groups B2 and B1 (in that order), while lower resistance levels were detected among isolates belonging to group D. The variation in the observations from the various studies could be attributed to the different geographical locations and to the origins of the *E. coli *isolates present at the different sites.

Principal component analysis (PCA) (Figure 4) demonstrated that the prevalence of antibiotic-resistant *E. coli* isolates was associated with the different sites, and the highest prevalence of AR was observed in *E. coli* isolates from the S2 site. Site S2 also showed the highest MDR index (0.31). This finding is probably due to its proximity to Marjan for Internal Medicine and Cardiology Hospital, where there is direct wastewater discharge to the S2 site. ARGs in the wastewater could be further disseminated by river flow from the S2 to the S3 site, as demonstrated by the prevalence of *E. coli* isolates resistant to last resort antibiotics, such as imipenem. Detection of cephalosporins, penicillins, and aminoglycosides resistance at the S1 site (located near agricultural lands) is probably related to their intensive use in agricultural practices and veterinary medicine. This study provides the first data regarding the prevalence of AR among the different phylogenetic groups in *E. coli* isolates recovered from the Al-Hillah River. Phylogenetic groups A and B2 possessed high AR that could enhance the resistance in the aquatic environment. A high rate of AR was mainly observed at the S2 site, which is located in an area of intensive hospital discharge. Overall, these findings reveal the importance of surface water as a reservoir for the dissemination of ARGs in natural aquatic environments.

## 5. Conclusion

The distribution of antibiotic-resistant *E. coli* isolates over various phylogenetic groups in the Al-Hillah River could be a public health risk.

## Figures and Tables

**Table 1:** Bacteriological parameters of the Al-Hillah River in three sampling sites.

Sites	BOD (mg/L)	TC *∗* CFU/100 mL (CFU × 10^3^)	EC *∗* CFU/100 mL (CFU × 10^3^)	E CFU/100 mL (CFU × 10^3^)
S1	4.15 ± 0.03	3.6 ± 0–4.7 ± 0	3 ± 0–3.5 ± 0	0–1.9 ± 0
S2	3.7 ± 0	3.4 ± 0–4.6 ± 0	2.9 ± 0–3.4 ± 0	0–1.6 ± 0
S3	3.14 ± 0	3 ± 0–4.6 ± 0	2.8 ± 0–3.3 ± 0	0–1.3 ± 0
Standard limits	**<4**	**200**	**126**	**35**

TC, total coliform; EC, *Escherichia coli*; E, Enterococci. Maxima and minima bacterial counts obtained from three sampling sites. Statistically significant correlation coefficients with *p* ≤ 0.05. All analyses from the three sampling sites were performed in triplicate and the standard deviations were less than 1.5% of averages.

## Data Availability

The data used to support the findings of this study are included within the supplementary information file.

## Supplementary Materials

Supplementary Materials Appendix 1: phylogenetic group PCR: sixty one isolates of *E. coli* were typed to one of the four major *E. coli* phylogenetic groups (A, B1, B2, D) according to Clermont et al. via a polymerase chain reaction-based assay (PCR) using the genetic markers *chuA* and *yjaA* and the TspE4.C2 DNA fragment. Appendix 2: language editing certificate.
