# Supplementary material for: Antibiotic Resistance Patterns of Diverse Escherichia coli Phylogenetic Groups Isolated from the Al-Hillah River in Babylon Province, Iraq
Source: ScientificWorldJournal. 2019 Sep 2;2019:5927059. doi: 10.1155/2019/5927059 (PMC6745164; doi:10.1155/2019/5927059)
Supplement: Supplementary Materials — Appendix 1: phylogenetic group PCR: sixty one isolates of E. coli were typed to one of the four major E. coli phylogenetic groups (A, B1, B2, D) according to Clermont et al. via a polymerase chain reaction-based assay (PCR) using the genetic markers chuA and yjaA and the TspE4.C2 DNA fragment. Appendix 2: language editing certificate. [file 5927059.f1.pdf]

Supplementary Materials

Appendix 1

Sixty one isolates of *E. coli* were typed to one of the four major *E. coli* phylogenetic groups (A, B1, B2, D) according to Clermont *et al*. via a polymerase chain reaction-based assay (PCR) using the genetic markers *chuA* and *yjaA* and the TspE4.C2 DNA fragment.

Appendix 2

Language Editing Certificate

Appendix 1

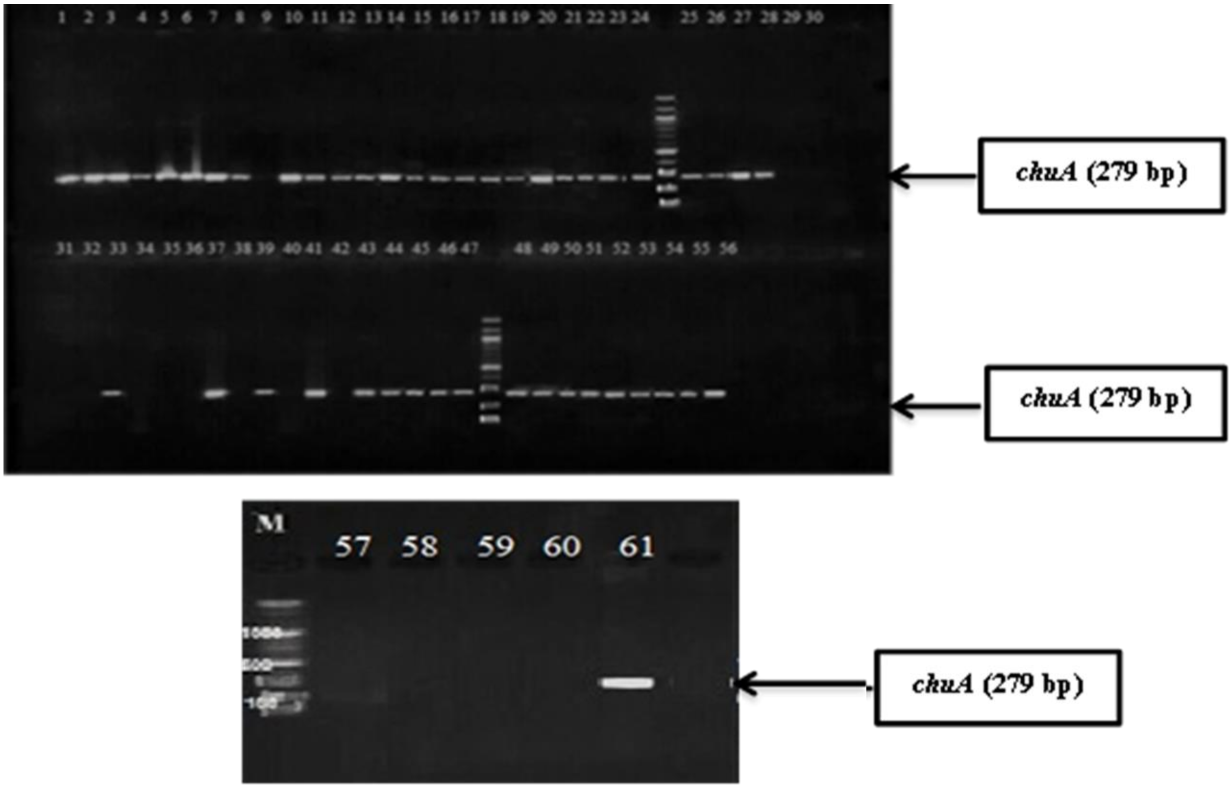

Figure 1: Agarose gel electrophoresis of PCR products derived from *chuA* genetic marker of 61 *E. coli* isolates. *chuA* (279 bp) marker codes for ChuA (hem uptake protein). Lane M is 1Kb DNA Ladder, lanes 1 to 61 are *E. coli* isolates. Samples were amplified on 1.5% agarose gel and isolates are labeled at the top of the figure 1.

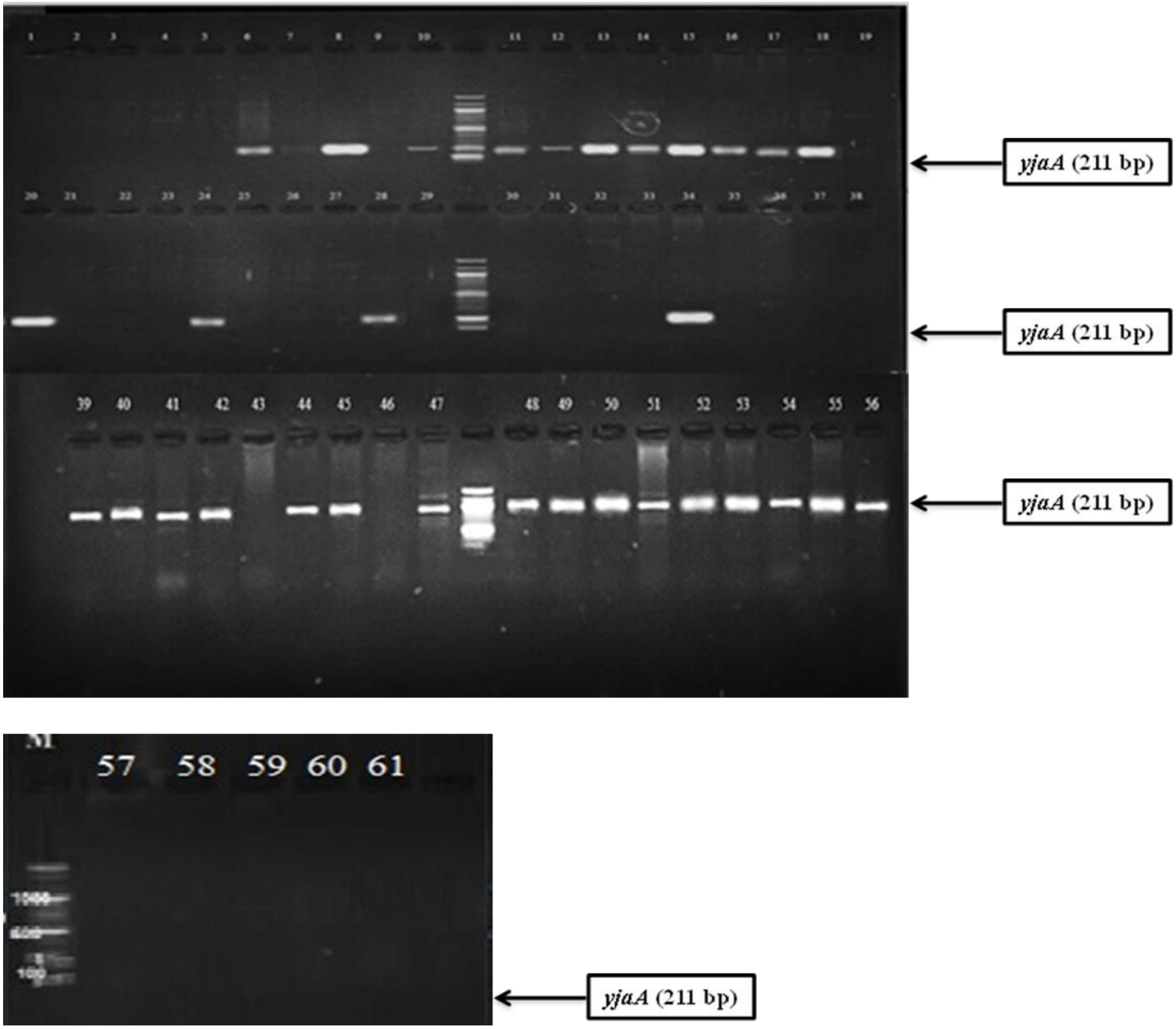

Figure 2: Agarose gel electrophoresis of PCR products derived from *yjaA* genetic marker of 61 *E. coli* isolates. *yjaA* (211 bp) marker codes for YjaA (stress induced protein). Lane M is 1Kb DNA Ladder, lanes 1 to 61 are *E. coli* isolates. Samples were amplified on 1.5% agarose gel and isolates are labeled at the top of the figure 2.

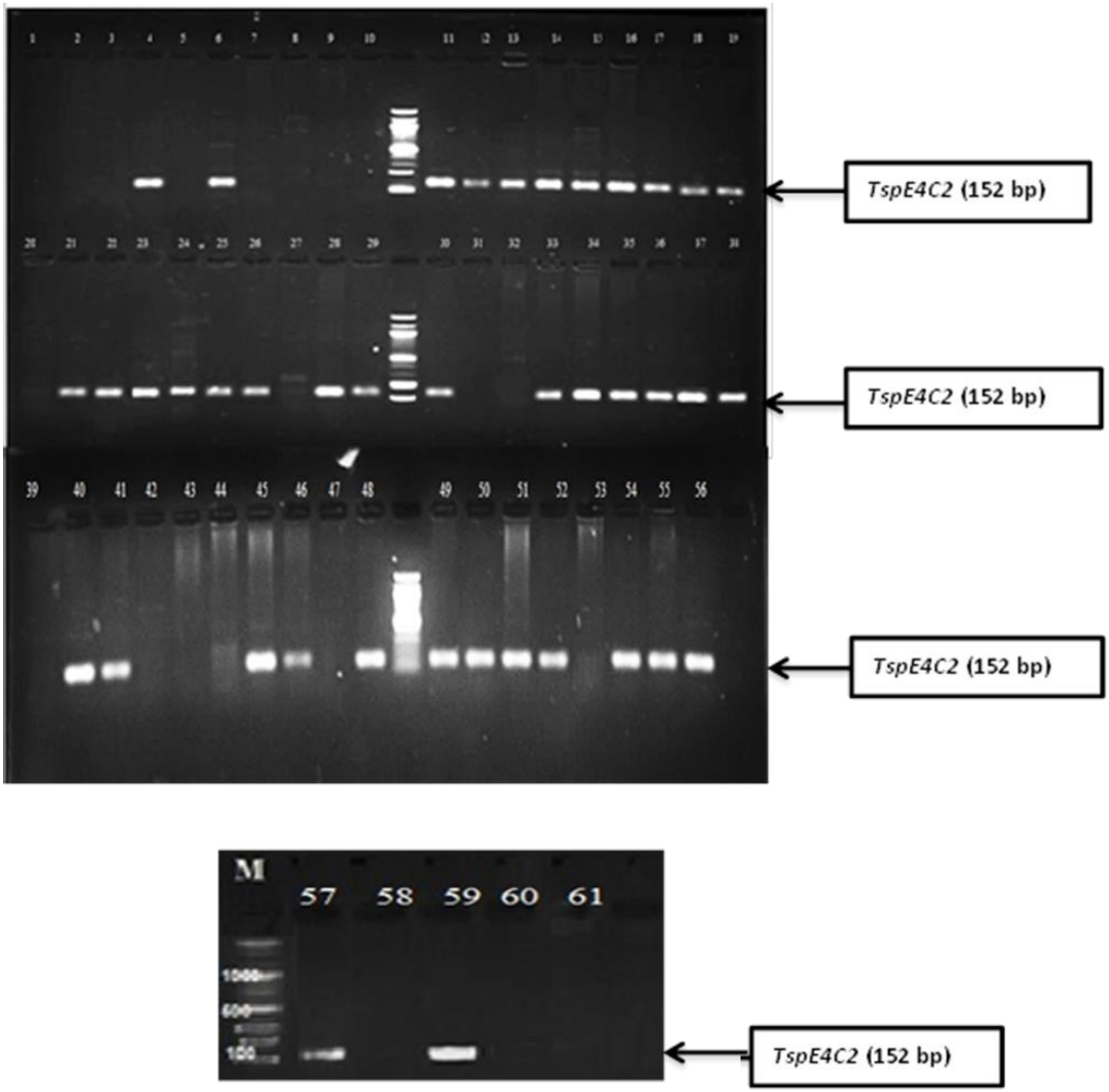

Figure 3: Agarose gel electrophoresis of PCR products derived from *TspE4C2* genetic marker of 61 *E. coli* isolates. *TspE4C2* (152 bp) marker codes for TSPE4C2 (part of lipase esterase). Lane M is 1Kb DNA Ladder, lanes 1 to 61 are *E. coli* isolates. Samples were amplified on 1.5% agarose gel and isolates are labeled at the top of the figure 3.

Appendix 2 (Language editing Certificate)

ELSEVIER
Language Editing Services

Registered Office:
Elsevier Ltd
The Boulevard, Langford Lane,
Kidlington, OX5 1GB, UK.
Registration No. 331566771

To whom it may concern

The paper "Antibiotic Resistance Patterns of Diverse Escherichia coli Phylogenetic Groups Isolated From Al-Hillah River, Babylon Province, Iraq" by Mourouge Saadi Abbas Alwash was edited by Elsevier Language Editing Services.

Kind regards,

Elsevier Webshop Support

(This is a computer generated advice and does not require any signature)
